# Source analysis of short and long latency vestibular-evoked potentials (VsEPs) produced by left vs. right ear air-conducted 500 Hz tone pips

**DOI:** 10.1016/j.heares.2014.03.006

**Published:** 2014-06

**Authors:** N.P.M. Todd, A.C. Paillard, K. Kluk, E. Whittle, J.G. Colebatch

**Affiliations:** a Faculty of Life Science, University of Manchester, Manchester M13 9PL, UK; b School of Psychological Sciences, University of Manchester, Manchester M13 9PL, UK; c Prince of Wales Clinical School and Neuroscience Research Australia, University of New South Wales, Randwick, Sydney, NSW 2052, Australia

## Abstract

Todd et al. (2014) have recently demonstrated the presence of vestibular dependent changes both in the morphology and in the intensity dependence of auditory evoked potentials (AEPs) when passing through the vestibular threshold as determined by vestibular evoked myogenic potentials (VEMPs). In this paper we extend this work by comparing left vs. right ear stimulation and by conducting a source analysis of the resulting evoked potentials of short and long latency. Ten healthy, right-handed subjects were recruited and evoked potentials were recorded to both left- and right-ear sound stimulation, above and below vestibular threshold. Below VEMP threshold, typical AEPs were recorded, consisting of mid-latency (MLR) waves Na and Pa followed by long latency AEPs (LAEPs) N1 and P2. In the supra-threshold condition, the expected changes in morphology were observed, consisting of: (1) short-latency vestibular evoked potentials (VsEPs) which have no auditory correlate, i.e. the ocular VEMP (OVEMP) and inion response related potentials; (2) a later deflection, labelled N42/P52, followed by the LAEPs N1 and P2. Statistical analysis of the vestibular dependent responses indicated a contralateral effect for inion related short-latency responses and a left-ear/right-hemisphere advantage for the long-latency responses. Source analysis indicated that the short-latency effects may be mediated by a contralateral projection to left cerebellum, while the long-latency effects were mediated by a contralateral projection to right cingulate cortex. In addition we found evidence of a possible vestibular contribution to the auditory T-complex in radial temporal lobe sources. These last results raise the possibility that acoustic activation of the otolith organs could potentially contribute to auditory processing.

## Highlights

• AEPs recorded below and above VEMP threshold for left vs. right ear stimulation.
• Above VEMP threshold expected short- and long-latency VsEPs observed.
• Short-latency VsEPs at 10 ms in infra-ocular and posterior leads.
• Longer-latency VsEPs characterised by N42/P52 in central leads, followed by N1.
• Source analysis indicates recruitment of cingulate vestibular cortex.

## 1 Introduction

Acoustic sensitivity of the human vestibular system has long been established and can be demonstrated by means of evoked electromyographic (EMG) signals (Bickford et al., 1964). Such EMG responses can be measured either from muscles of the neck, e.g. the sternocleidomastoid muscle, reflecting the vestibular-collic reflex pathways (the vestibular evoked myogenic potential or VEMP (Colebatch et al., 1994)) or from extra-ocular eye muscles, reflecting activation of the vestibular ocular reflex pathways (ocular VEMP or OVEMP (Rosengren et al., 2005; Todd et al., 2007)). Although the neck response is often now referred to as a cervical VEMP (or CVEMP), in the rest of this text we use the original acronym ‘VEMP’.

In the last decade evidence has accumulated from electroencephalographic (EEG) studies in humans that vestibular receptors may also contribute to sound evoked potentials of central origin. Following a study by de Waele et al. (2001), who showed the existence of short-latency potentials (8–15 ms) in response to electrical stimulation, Todd et al. (2003) demonstrated a similar response to 500 Hz bone-conducted (BC) sound. These acoustically evoked short-latency vestibular evoked potentials (VsEPs) were confirmed to have vestibular origin as they were absent in avestibular patients but present in deaf subjects with intact VEMPs (Rosengren and Colebatch, 2006). (Unlike AEPs for VsEPs there is no well-established “brainstem” response hence really only two epochs are recognized. The term “short latency VsEPs” has become established in the literature as referring to early responses dominated by the vestibular reflexes. These overlap in time with both ABR and MLR AEPs.) Later Todd et al. (2008) used source analysis to confirm that the short-latency VsEPs produced by air-conducted (AC) and BC sound are dominated by the pathways underlying the vestibular-ocular reflex, but also suggested activity in frontal cortex. More recently McNerney et al. (2011) used an alternative source analysis method to suggest that a wider range of vestibular cortical areas contribute to the short-latency potentials activated by sound.

Most recently Todd et al. (2014) provided evidence that in addition to short-latency effects there were likely vestibular contributions at longer latencies. These were recognized by systematic changes that take place in morphology and in the intensity dependence of the responses in passing through the vestibular threshold. Of particular interest was a medium-latency deflection, labelled N42/P52, which exhibited a change in slope and latency function, and was absent in an avestibular patient. The long-latency auditory evoked potential (LAEP) N1 also showed some changes in passing through the VEMP threshold. A source analysis indicated a possible contribution of cingulate cortex to both the N42 and N1, as well as temporal lobe, cerebellar and other sub-cortical sources. This study was, however, limited to the left ear only. We wished, therefore, in the present study to extend this work to both left and right ears and to investigate any effects of ear or laterality of vestibular dependent changes in sound evoked potentials, and in particular the N42/P52 and N1 waves, especially in light of lateralisation shown for vestibular stimulation in imaging studies (Dieterich et al., 2003; Janzen et al., 2008; Schlindwein et al., 2008; Lopez et al., 2012).

## 2 Material and methods

### 2.1 Subjects

Ten healthy subjects were selected for this study (mean age = 27.5; SD = 7.21; 3 females and 7 males). All subjects were first screened for any neurological, vestibular or hearing impairment. Prior to any testing, all participants gave written informed consent according to the Declaration of Helsinki. The University of Manchester Ethics Committee approved the study.

### 2.2 Stimuli

The experimental stimuli employed for obtaining vestibular responses were AC 2-ms, 500-Hz, single cycle tone pips. AC stimuli were delivered by insert earphones (3A insert earphone, E-A-RTone Gold, Guymark UK Limited). The maximum input voltage resulted in a maximum output on the amplifier equivalent to a peak SPL (pkSPL) of 136 dB re 20 μPa (as measured by the LLpk parameter with linear frequency weighting). Stimulus calibration was carried out using a GRAS IEC711 Coupler (RA0045) and a pressure-field microphone (Model 4134) with a 2260 Investigator (Brüel and Kjaer, Naerum, Denmark). The stimuli were generated using customised software with a laboratory interface (power1401, Cambridge Electronic Design, Cambridge, UK) and a commercial or custom amplifier.

### 2.3 Auditory thresholds

Audiograms were obtained for both ears using an Amplivox audiometer (Amplivox Ltd, UK) with Telephonics TDH 49 earphones (Telephonics Corp., Farmingdale, NY, USA). Each subject satisfactorily achieved pure tone air conduction thresholds of ≤20 dB HL at 125, 250, 500, 1000, 2000, 4000 and 8000 Hz bilaterally, according to British Society of Audiology (BSA) (2011) recommended procedures. The subjects had no history of otological or neurological pathology.

Absolute auditory threshold measurements were performed using PsyLab (v2.0, Hansen, 2006) using 3-alternative forced choice (3AFC), one-up two-down adaptive method to track the 79.4% point on the psychometric function (Levitt, 1971). The signal, i.e. 2-ms, 500-Hz, single-cycle tone-pip, was randomly presented to the subject in one of the three intervals and delivered unilaterally through insert earphones (3A insert earphone, E-A-RTone Gold, Guymark UK Limited). The initial signal level was set to 81 dB pkSPL; this was reduced by 4 dB after two successive correct responses and increased by 4 dB after an incorrect response. After four reversals the measurement phase began and the step size was reduced to 1 dB. The threshold was taken as an average of the last four reversals.

### 2.4 Vestibular thresholds

Vestibular thresholds were obtained by means of VEMPs. Subjects were tested lying supine on a couch, with the backrest approximately tilted to 30–45° from the horizontal, and required to lift their heads against gravity to activate the sternocleidomastoid (SCM) muscles. Surface EMG was measured from the ipsilateral SCM using self-adhesive Ag/AgCl electrodes. Active surface electrodes were placed over the middle of the SCM muscle belly and were referred to electrodes placed on the medial clavicle. EMG was amplified, bandpass filtered (5 Hz–1 kHz) and sampled using a Power1401 interface (CED Ltd., Cambridge, UK). The EMG was sampled at a rate of 5 kHz, starting 10 ms before to 80 ms following stimulus onset, and averaged. Stimuli were delivered by insert earphones (3A insert earphone, E-A-RTone Gold, Guymark UK Limited). Up to 200 stimuli were presented at a rate of about 6 Hz.

VEMP thresholds (V_T_) were determined for each subject by reducing the stimulus intensity in 5 dB steps over successive trials and were defined as the smallest intensity at which a VEMP could be produced in at least two trials. The procedure was performed for left and right sides of stimulation independently and randomly across subjects.

### 2.5 VsEPs

VsEPs were recorded with subjects comfortably seated with their gaze directed straight ahead to a screen displaying silent movies at a viewing distance (about 70 cm). AC pips were presented with interstimulus intervals randomly varying between 600 and 1000 ms, up to a total of 400 stimuli per trial. Evoked potentials (EPs) were recorded for three intensities: maximal intensity (136 dB pkSPL), 0 re V_T_ and −12 dB re V_T_. Left and right ears were stimulated separately and randomly across subjects. EEG was recorded using a 64-channel EEG system (Biosemi, Inc., USA). Additional electrodes were also placed below each eye (i.e. infra-ocular electrodes, IO1 and IO2), at deep frontal (F9 and F10) and at ear-lobe locations (A1 and A2). Electrode offset (i.e. running average of the voltage measured between CMS and each active electrode) was maintained below 20 μV. Recordings were made with a band-pass of between 0.16 Hz and 1 kHz. Artefact elimination, epoching and averaging of EPs were carried out using the BESA 5 software. Epochs were 350 ms in length, from 50 ms before to 300 ms following the stimulus onset. After collection, EPs were filtered at 1–200 Hz and referenced either to linked ear-lobe electrodes or to an average reference using Scan software (v4.3, Neuroscan, USA). Amplitudes were measured at responses peaks. Although all BESA was done using the average reference we retained linked-ears for electrode measurements as this is a standard montage, including in the clinical setting.

### 2.6 Source analyses

BESA software (version 5.1 MEGIS Software GmbH, Germany) was used for dipole modelling. The standard four-shell elliptical head approximation was employed with the following parameters. The radial thickness of the head, scalp, bone and CSF were 85, 6, 7 and 1 mm, respectively, with conductivities set to 0.33, 0.33, 0.0042 and 1.0, respectively. Prior to conducting the source analysis changes in the global field power with intensity were also evaluated in order to determine the appropriate fitting epoch. After extensive trials it was found appropriate to model the entire epoch from 7 to 235 ms covering short- and long-latency effects. We adopted a modelling strategy from previous occasions of using pairs of regional sources and dipoles (Todd et al., 2008, 2014). This approach was arrived at after extensive modelling using different strategies. Ocular sources and temporal lobe sources are ubiquitous for the stimuli employed and two pairs locate without fail to these areas, irrespective of starting conditions. Regional sources are appropriate to model the complexity of the (known) activation of the bilateral extra-ocular eye muscles (EOM) in conjunction with the retinal corneal dipole (RCD) associated with eye movement, and for activity in bilateral temporal cortex, which includes independent radial and tangential components. For the additional dipole pair sources no constraint was applied other than symmetry, the starting point for these being determined by previous solutions indicating anterior and posterior regions, with the ocular and temporal sources starting from their original positions from the lower order solutions.

BESA was the analytical approach of choice as although methods such as Low Resolution Electromagnetic Tomography (LORETA) provide an alternative approach with different assumptions, BESA is uniquely able to model both EEG and (extracranial) EMG sources. LORETA solutions are also limited to cortical grey areas, and thus in addition to misinterpreting signals from EOG or extra-ocular muscle sources, it would fail to detect, or misinterpret signals from sub-cortical sources such as the cerebellum or thalamus.

### 2.7 Statistical analysis

ANOVA were carried out on short latency amplitude measurements on two recording regions of interest, using side of stimulation as a factor, i.e. the infra-ocular leads IO1 and IO2, and the inion related leads Iz, PO7 and PO8. The PO leads were chosen as the scalp analysis indicated that the expected short-latency effects were maximal at these sites. As the ocular and inion related channels showed a similarity in patterns of response, a joint analysis of the IO and PO channels was also conducted. For the longer latency response two regions of interest were: fronto-central leads Fpz and FCz, and lateral fronto-central leads FC3 and FC4. The FC leads were chosen as the N1 is maximal at these sites.

## 3 Results

### 3.1 Thresholds

VEMP thresholds (V_T_) were recorded in 10 healthy subjects, with a mean (SD) threshold of 108.6 (5.2) dB and 109.5 (6.9) dB pkSPL for left and right AC stimulation. Absolute auditory thresholds were 24.5 (3.1) dB and 26.0 (4.8) dB pkSPL for left and right AC stimulation, respectively. Combined together these are equivalent to 84.1 and 83.5 dB sensation level (SL), similar values to those found previously.

### 3.2 Properties of the averaged electroencephalography (EEG)

**Fig. 1:**
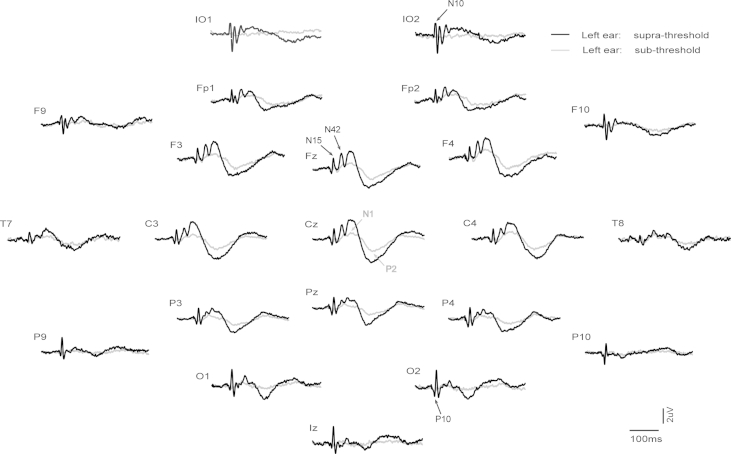
Grand means of evoked potentials produced by 500 Hz, 2 ms pips from left ear stimulation in 10 healthy subjects. For each electrode location the two traces show the supra-threshold (maximal intensity) vs. sub-threshold (−12 dB re V_T_) conditions as black and grey traces respectively. All electrodes are referred to the auricle leads.

**Fig. 2:**
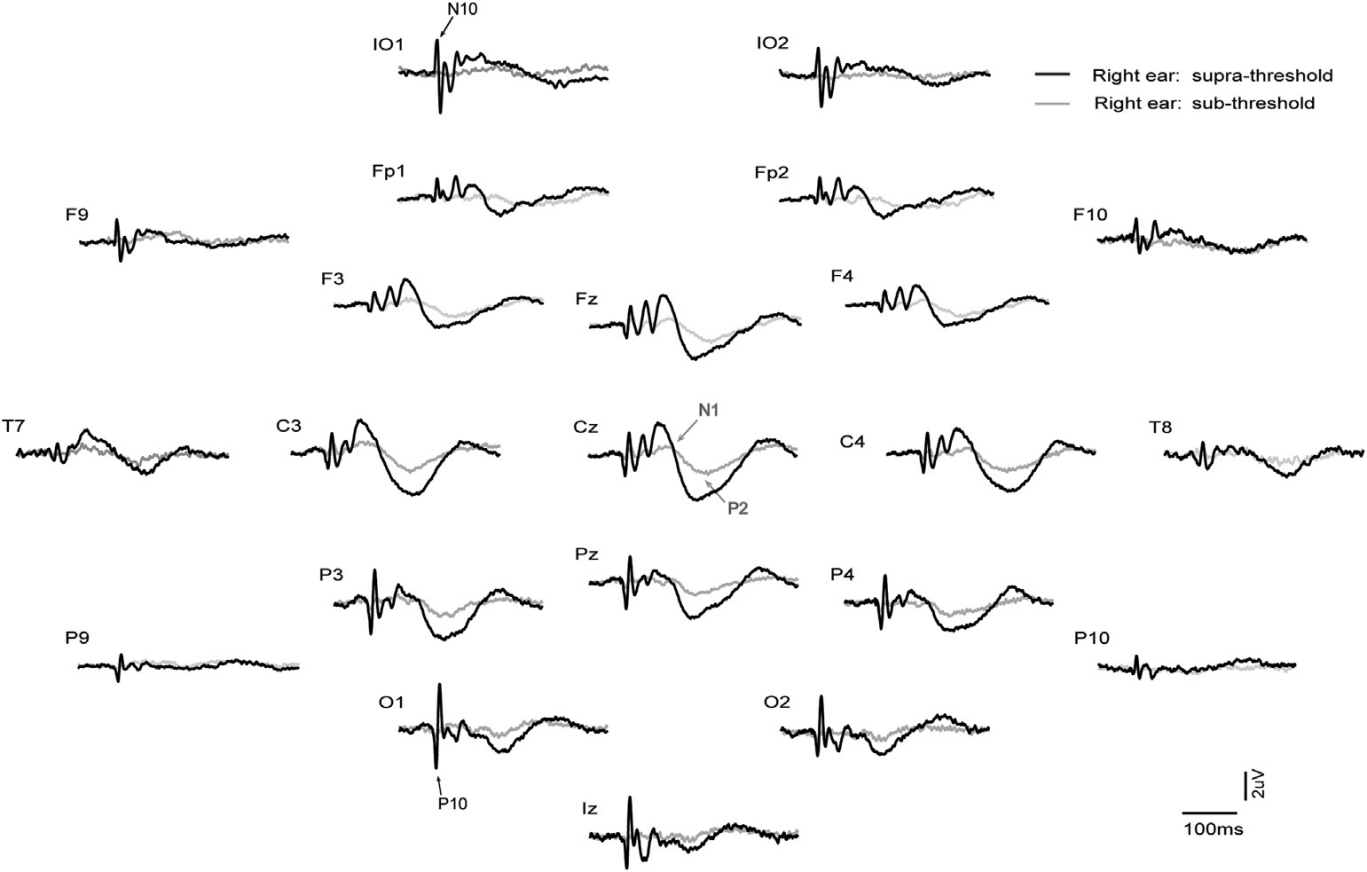
Grand means of evoked potentials produced by 500 Hz, 2 ms pips from right ear stimulation in 10 healthy subjects. For each electrode location the two traces show the supra-threshold (maximal intensity) vs. sub-threshold (−12 dB re V_T_) conditions as black and grey traces respectively.

**Fig. 3:**
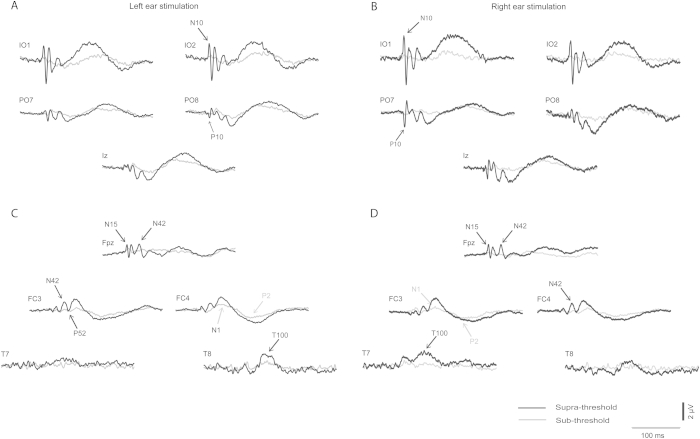
Grand means of evoked potentials produced by 500 Hz, 2 ms pips in selected peripheral leads which illustrate short-latency effects from (A) left ear and (B) right ear stimulation at supra-threshold (black) vs. sub-threshold (grey) intensities. For the same stimulus conditions grand means are also illustrated in selected fronto-central and lateral leads which illustrate long-latency effects from (C) left and (D) right ear stimulation at supra-threshold (black) vs. sub-threshold (grey) intensities.

**Fig. 4:**
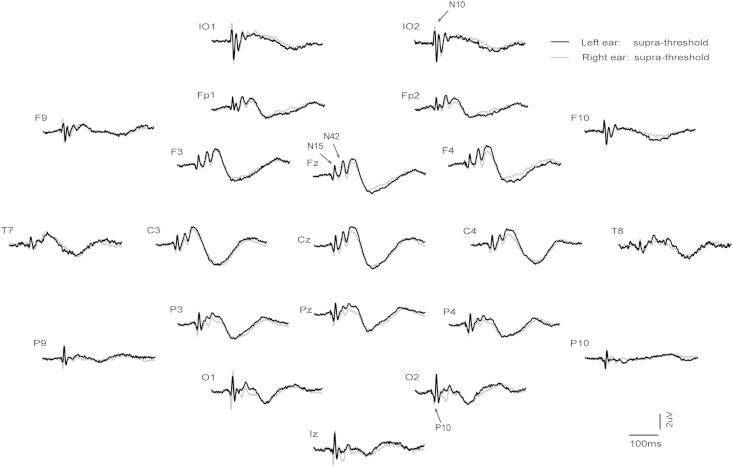
Grand means of evoked potentials produced by 500 Hz, 2 ms pips at supra-threshold (maximal intensity) for left (black) vs. right ear (grey) stimulation.

**Fig. 5:**
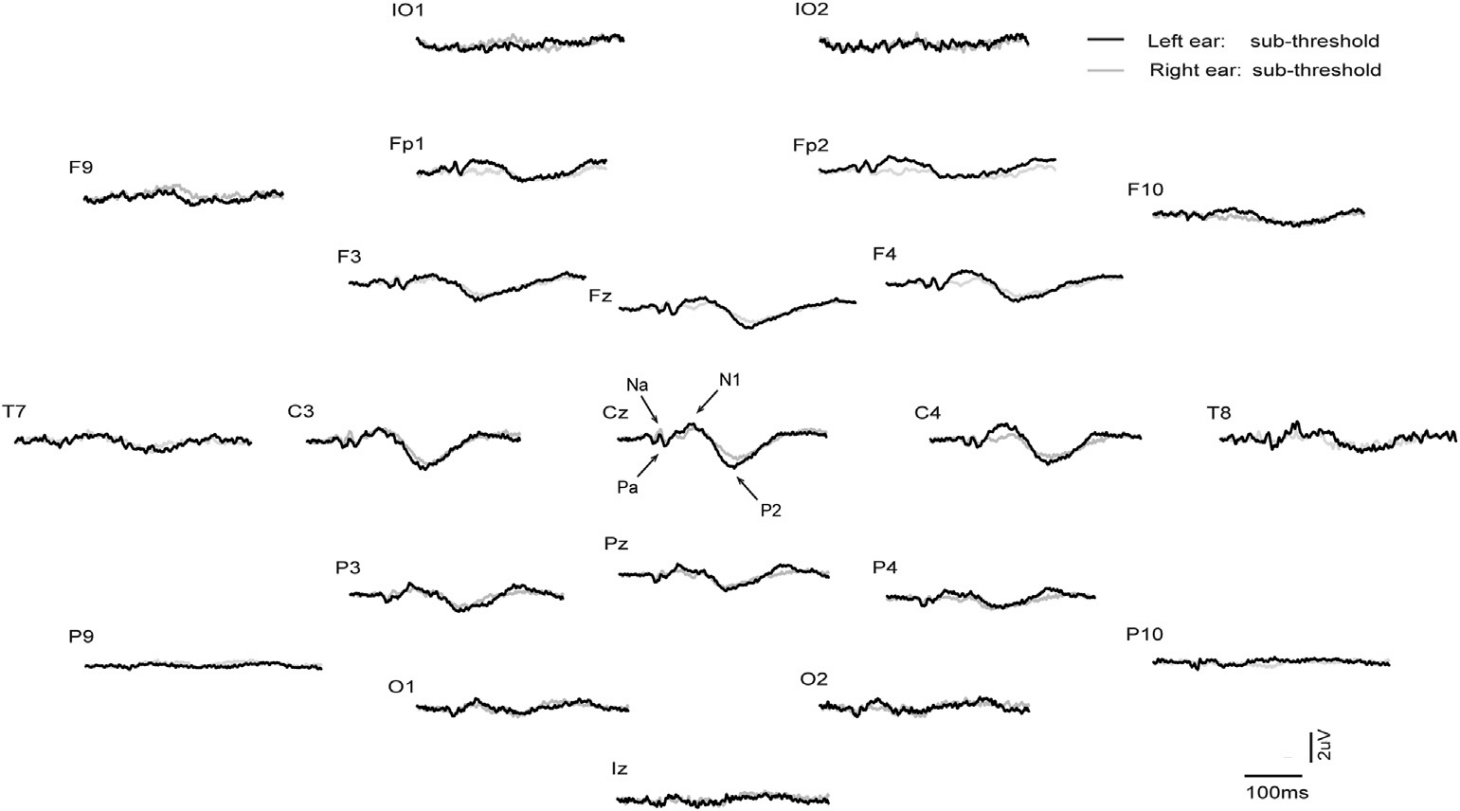
Grand means of evoked potentials produced by 500 Hz, 2 ms pips at the sub-threshold intensity (−12 dB re V_T_) for left (black) vs. right ear (grey) stimulation.

**Fig. 6:**
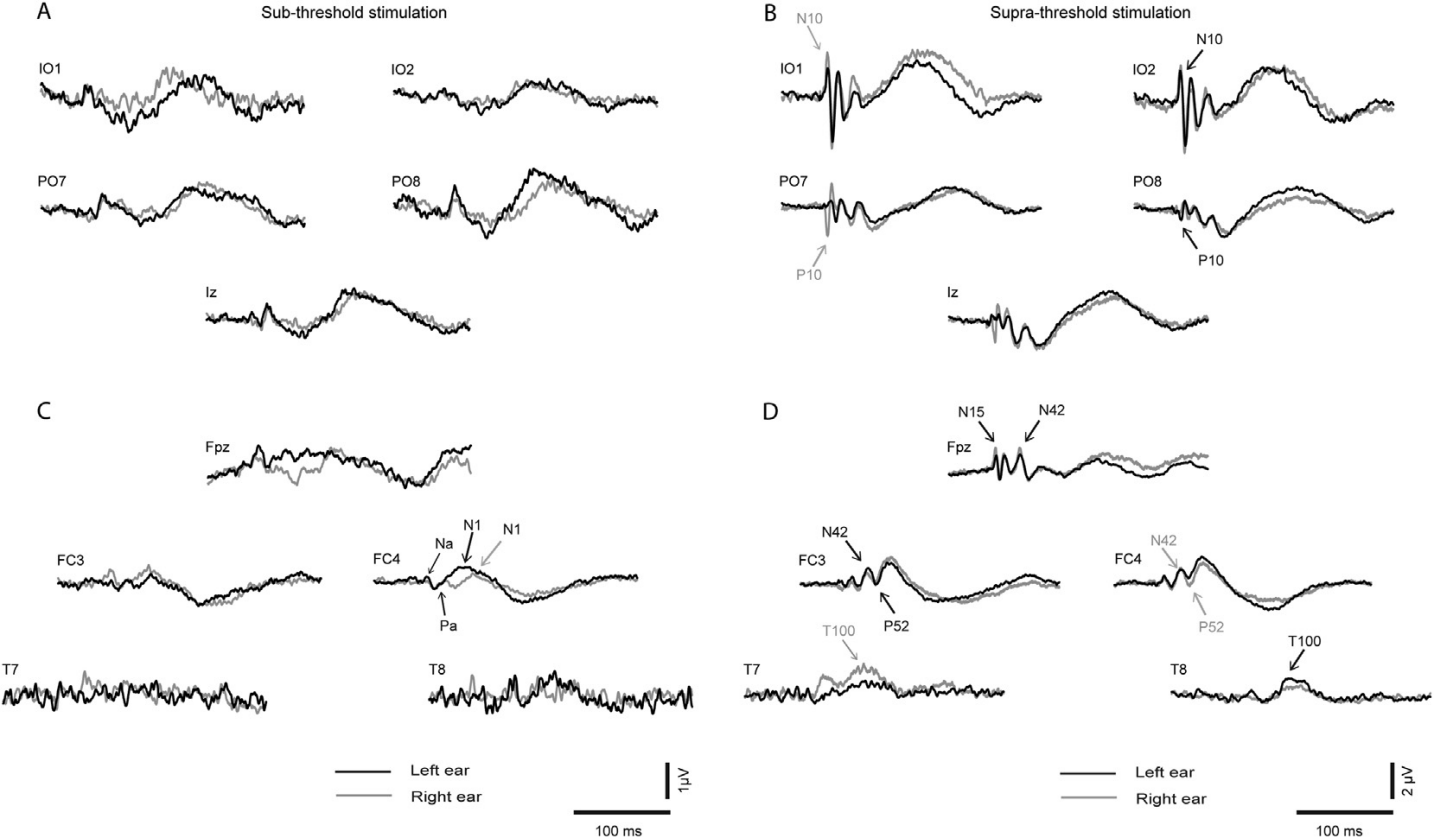
Grand means of evoked potentials in selected peripheral leads which illustrate short-latency effects produced by (A) sub-threshold (−12 dB re V_T_) and (B) supra-threshold (maximal intensity) for left (black) vs. right (grey) ear stimulation. For the same stimulus conditions grand means are also illustrated in selected fronto-central and lateral leads which illustrate long-latency effect produced by (C) sub-threshold and (D) supra-threshold intensities for left (black) vs. right (grey) ear stimulation.

Grand means for EPs produced by supra- vs. sub-threshold (−12 dB re. V_T_) intensities for left and right ear stimulation are shown in Figs. 1 and 2 respectively (with details for selected channels given in Fig. 3), and for left vs. right ear stimulation for supra- and sub-threshold intensities in Figs. 4 and 5 (with details for selected channels given in Fig. 6). As has been established for left ear stimulation (Todd et al., 2014), the sub-threshold condition shows a typical AEP pattern consisting of mid-latency (MLR) Na and Pa waves followed by the long latency (LAEP) N1 and P2 waves, well illustrated in channel Cz (Fig. 5). In contrast, the supra-threshold condition shows the expected changes in morphology. These are characterised by the short-latency waves, which have no auditory correlate, the OVEMP and inion related response N10 and P10 (Figs. 3A, B and 6B), and a later deflection, labelled N42/P52 followed by the LAEP N1 and P2 (Figs. 3C, D and 6D).

### 3.3 Differences in the averaged EEG with ear of stimulation

As noted above, the effects of ear of stimulation are illustrated in Figs. 4–6. The sub-threshold stimulation condition showed the expected absence of vestibular components in the infra-ocular and inion related leads (Fig. 5) and expected presence of AEPs in the central leads (Fig. 6C). This condition also displayed an asymmetry to left ear stimulation as previously described in the literature (Hine and Debener, 2007). In contrast the supra-threshold intensity exhibited VEMP-related responses in the infra-ocular electrodes and inion region (Fig. 6B). Of interest, the early wave patterns (N10/P10) in IO and PO leads appeared to be mirroring each other especially on the contralateral side. Within the central leads (Fig. 6D) there appeared to be a similar left ear advantage in the central and lateral frontal waves as for the sub-threshold case. In contrast for the VEMP-related responses (Fig. 6B) the largest amplitudes were recorded during right ear stimulation.

### 3.4 Statistical analysis of differences in amplitude: short latency effects

ANOVA on the IO1 and IO2 were carried out using three factors ear (L vs. R), wave (N10 or P17) and side, coded either for laterality (ipsi- vs. contra-) or eye (left vs. right). Although right ear stimulation produced larger responses in both eyes there were no significant effects nor any interactions (*p* > 0.05).

As visual inspection (Fig. 6) indicated that there was an ear or laterality effect in the inion response, we measured this at both PO7 and PO8 for the first positive (P10) and negative (N17) waves. No significant main effects of ear or laterality were obtained, but a two-way interaction between laterality and wave (*F*_(1,8)_ = 15, *p* < 0.01) indicated that there was a contralateral dominance for the initial P10, 0.8 vs. 1.6 μV, but not the N17.

Given the apparent mirroring in the IO and PO leads we also carried out a joint analysis of the N10/P17 and P10/N17 waves. These showed a similar pattern of significance as for the PO leads in isolation, i.e. there was a two-way interaction between laterality and wave (*F*_(1,8)_ = 8.3, *p* < 0.01), indicating contralateral dominance for the first wave (P10/N10), 1.3 vs. 1.7 μV. In addition there were also main effects of wave, with the second being larger than the first, 1.5 vs. 2.1 μV, and main effect of electrode, with the IO leads giving larger amplitudes than the PO leads, 2.3 vs. 1.3 μV.

ANOVA of Na/N15 amplitude at Fpz with ear (L vs. R) and intensity (sub- vs. supra-threshold) factors yielded an effect of intensity only (*F*_(1,8)_ = 24, *p* < 0.005), as would be expected.

### 3.5 Statistical analysis of differences in amplitude: long latency effects

ANOVA of the middle latency N42 potential with factors of electrode (Fpz vs. FCz), intensity (Max vs. −12 dB) and ear, yielded a main effect of intensity (*F*_(1,8)_ = 108, *p* < 0.001), as would be expected, but no other main effects. There was however a three-way interaction between electrode, ear and intensity (*F*_(1,8)_ = 8.5, *p* < 0.05), indicating a left ear advantage for the N42, but only apparent at the supra-threshold intensity and at FCz, 2.5 vs. 1.7 μV. With the peak–peak amplitude of the N42/P52 deflection, however, in addition to expected intensity effect, the ear advantage switched to the right ear as a main effect (*F*_(1,8)_ = 7.8, *p* < 0.05), with no interactions, but the magnitude of the difference was small, 1.3 vs. 1.5 μV.

ANOVA on the N1 and P2 amplitudes at FCz with factors, intensity, ear (L vs. R) and wave (N1 vs. N2) yielded the expected main effect of intensity (*F*_(1,8)_ = 33, *p* < 0.001), but also a main effect of ear, indicating a distinct left ear advantage (*F*_(1,8)_ = 18, *p* < 0.005), 2.8 vs. 2.2 μV. There were no significant interactions. A four-way ANOVA on the N1 and P2 amplitudes at FC3 and FC4, with factors of laterality (ipsi- vs. contra-), ear (L vs. R), intensity and wave (N1 vs. P2) was also conducted. This yielded main effects of laterality (*F*_(1,8)_ = 42, *p* < 0.001), ear (*F*_(1,8)_ = 13, *p* < 0.01) and intensity (*F*_(1,8)_ = 28.5, *p* < 0.005) but also an ear by intensity interaction (*F*_(1,8)_ = 11, *p* < 0.05). These indicated a contralateral advantage (2.1 vs. 2.4 μV) and an overall left ear advantage (2.5 vs. 2.0 μV), but the left ear advantage was significantly enhanced at the supra-threshold intensity (3.3 vs. 2.6 μV). If coded by electrode (FC3 vs. FC4) rather than laterality (ipsi- vs. contra-) there was no effect of electrode, but there was an electrode by ear interaction (*p* < 0.001) so that the left ear advantage was strongest in FC4 on the right hemisphere (2.0 vs. 2.6 μV).

### 3.6 Source analyses

**Fig. 7:**
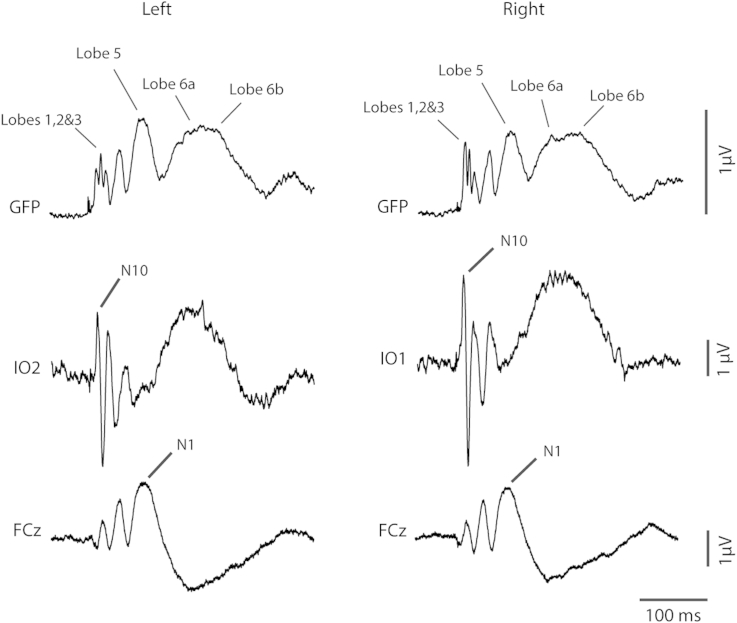
Global field power vs. FCz and IO leads for left vs. right stimulation. Lobes 1,2 and 3 correspond to the short-latency VsEPs, lobe 4 to the N42 and lobes 5 to the N1. Lobe 6 includes contributions from the T100 (indicated as 6a) and P2 (indicated as 6b).

Fig. 7 illustrates the structure of the global field power (GFP) for both left and right supra-threshold cases. Consistent with previous observations it was characterised by six lobes, three of short-latency, the first of which included effects of the N10/P10 and N15 measured in infra-ocular and posterior leads, and three of longer latency corresponding to the N42, N1 and P2 measured in central leads. The sixth lobe is quite broad with what appears to be early and late sub-components, which we label as lobes 6a and 6b. Source analysis was conducted over the whole epoch from 7 to 235 ms covering all six lobes. The temporal sources were modelled as a regional source for the fitting and then the components adjusted so that components 1 and 2 were tangential and component 3 radial (Näätänen and Picton, 1987; Scherg et al., 1989). For clarity the second tangential component is not included in the description.

Tables 1and 2 show respectively the outcomes of source modelling for both sub- and supra-threshold conditions. For both cases one pair of regional sources locate to bilateral insula cortex with a residual variance (RV) for the supra-threshold case of about 8% and for the sub-threshold case of 11–16%. The addition of a second pair of regional sources then locate within the orbit and the insula pair move laterally to a superior temporal location. Thereafter the behaviour of addition of bilateral pairs of dipoles diverges. For both the left and right supra-threshold cases the first pair of dipoles locates to a cingulate area and the second to cerebellum, with the insula pair of regional sources migrating further laterally to superior temporal cortex. The left sub-threshold case follows the same pattern as the supra-threshold but for the right ear case the first pair of dipoles locates to frontal cortex and there is no four pair solution which is stable.

**Fig. 8:**
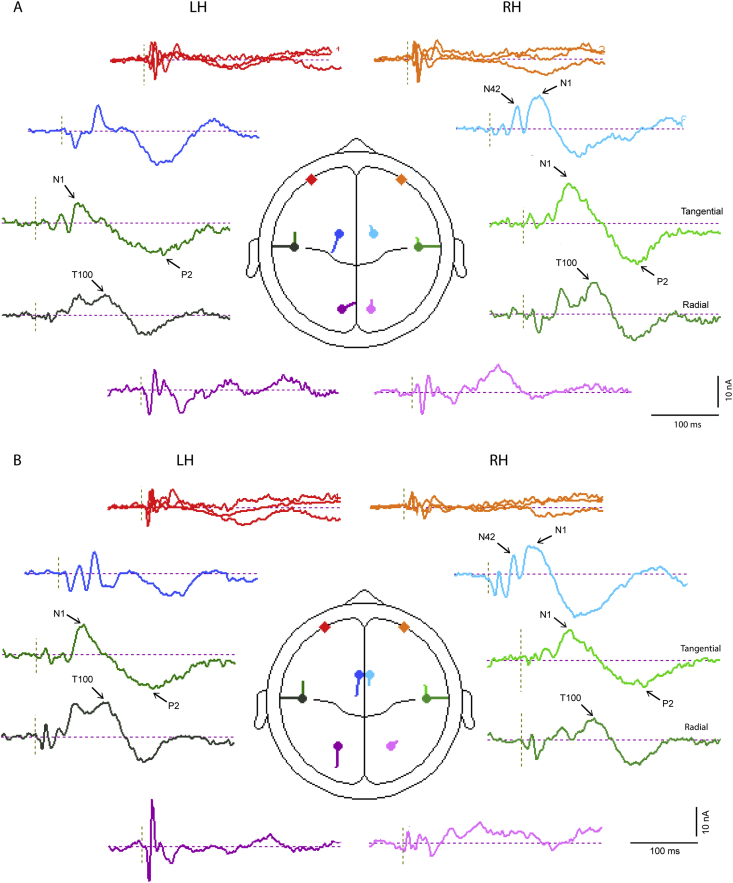
Source current waveforms for 8 pair BESA model of (A) left and (B) right ear stimulation at supra-threshold intensities. Source waveforms and locations for 4 pair model of left ear stimulation. Ocular sources red (left) and orange (right); cingulate sources dark (left) and light blue (right); temporal sources dark (left) and light green (right) (tangential and radial sources shown separately); and cerebellar sources dark (left) and light mauve (right). (For interpretation of the references to colour in this figure legend, the reader is referred to the web version of this article.)

**Fig. 9:**
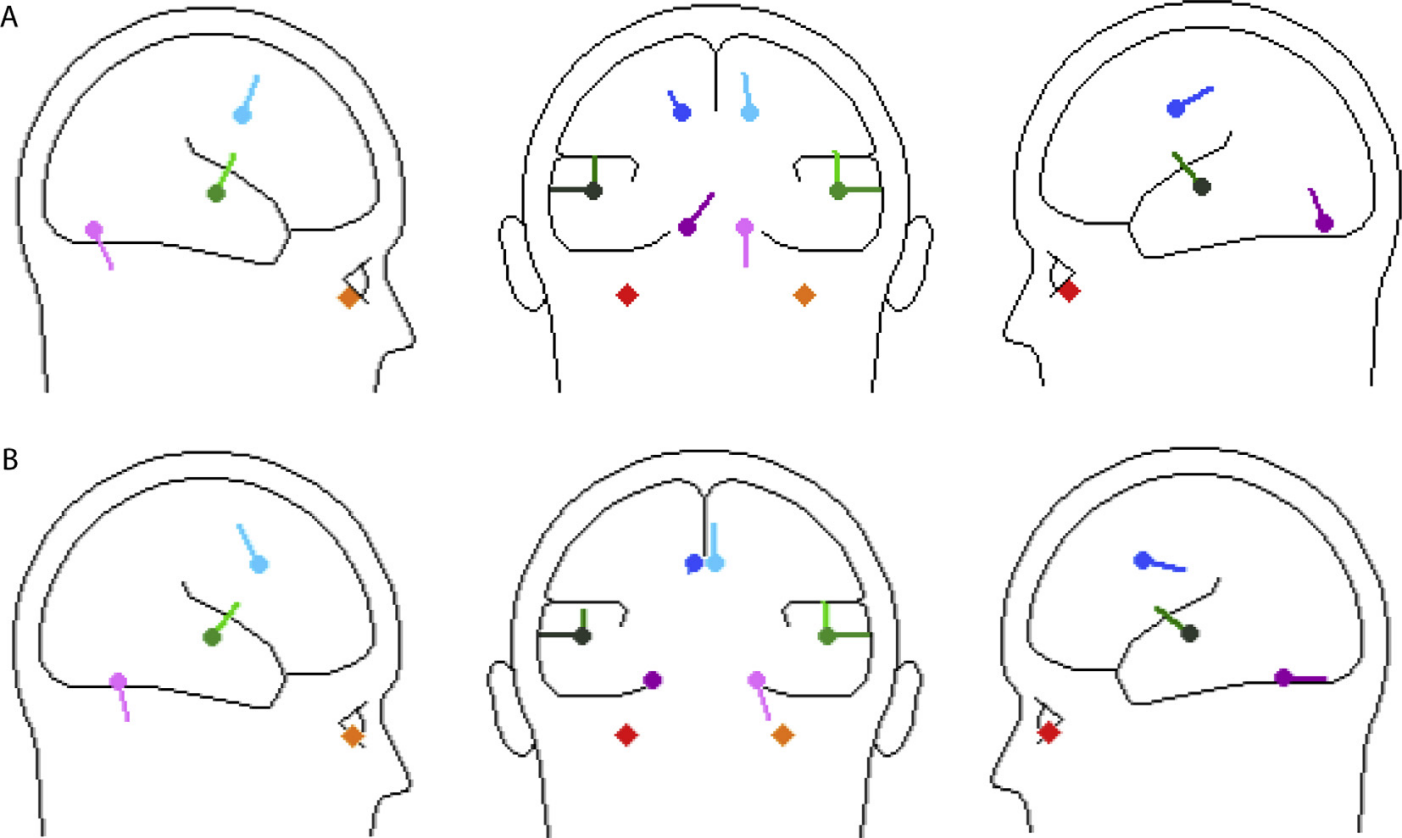
Sagittal and coronal views of source locations for 4 pair model of (A) left and (B) right ear stimulation.

In order to illustrate further the temporal pattern and divergences in behaviour, current source waveforms and transverse view locations are shown in Fig. 8 with the corresponding coronal and sagittal view locations given in Fig. 9. The source waveforms are shown in approximate location from anterior to posterior. The source current strengths are given in Table 3. Considering first the ocular and cerebellar sources, these show early bilateral activation corresponding with the short-latency VsEPs. An asymmetry is apparent in that the right ear stimulation produces a highly lateralised early cerebellar response (lobes 1 and 2) while for left ear stimulation the cerebellar response is more bilateral (see also Table 3). For the ocular sources the largest currents occur during the second lobe bilaterally for left ear stimulation. The cingulate sources show activation over the whole epoch but the largest currents occur during the later part of the epoch. For the N42 (lobe 4) the cingulate contribution is contralateral. However, for the N1 waves the right-hemisphere cingulate source is largest for both left and right ear stimulation (2 vs. 11 nA for left ear and 4 vs. 10 nA for right ear stimulation).

The last pair corresponds to the temporal cortex sources and in this case the tangential and radial components are shown separately. For left ear stimulation there is a well-defined asymmetry between the ipsilateral and contralateral tangential components at the latency of the N1 (about 74 ms) with the contralateral larger by a factor of about 3 (6 vs. 15 nA). In contrast the tangential N1 components for right ear stimulation are approximately equal (11 vs. 10 nA). The radial components show a quite different behaviour indicating a double peak with first at about 65 ms contributing to the onset of the N1 and the later peak at about 110, labelled T100, during onset of the sixth lobe with a polarity reversal at the latency of the P2 (Table 3). This component shows a well-defined contralateral dominance for both left and right ear stimulation.

**Fig. 10:**
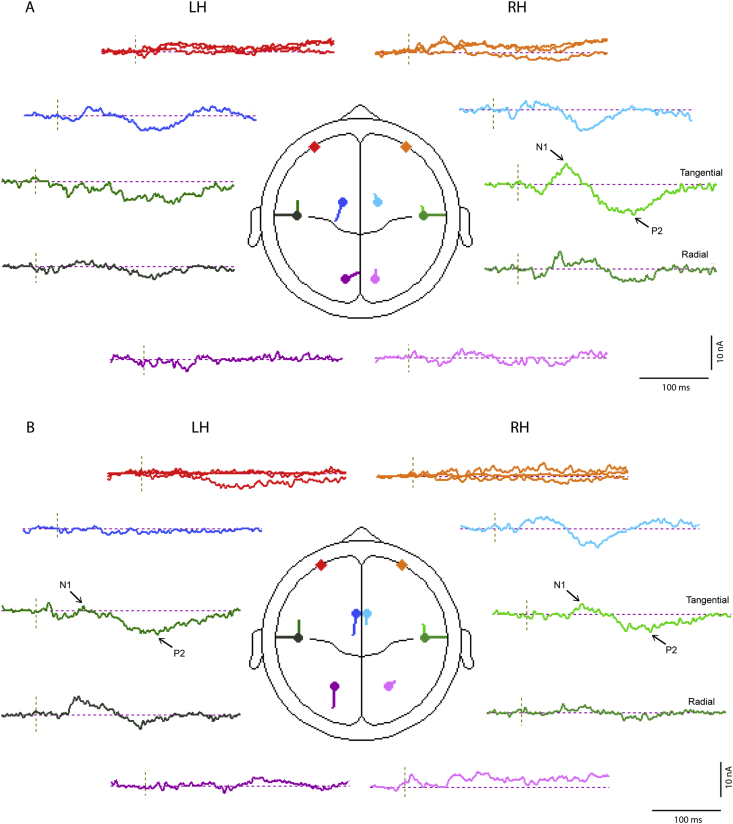
Source waveforms and locations for 4 pair model of (A) left and (B) right ear stimulation at supra-threshold intensity applied to the sub-threshold data.

As comparative solutions with 4 pairs could not be obtained for the sub-threshold cases we show in Fig. 10 the results of the source waveforms using the supra-threshold solutions. There is clearly no early activity in the ocular sources in the sub-threshold cases and the later activity corresponds to eye movements during the epoch. Similarly there is little activity in the cerebellar sources. For the cingulate sources there is activity at the latency of the P2 but little or no activity associated with the N1. As expected for the N1 the largest activity is found in the temporal sources. For left ear stimulation the N1 activation is strongly lateralised to the right hemisphere in the tangential component, with some activity in the contralateral radial component. For right ear stimulation the activity is more evenly distributed among the components on both sides for both N1 and P2, but again with some evidence of activity in the contralateral radial component. Although there is some evidence of the T100 in the radial component following the N1, it is small or inconsistent in the sub-threshold cases compared with the supra-threshold cases.

## 4 Discussion

The results above replicate short-latency effects reported in early studies, i.e. Todd et al. (2003, 2008), in demonstrating that when passing through the VEMP threshold a series of potentials, which have no auditory correlate, are observed. These are: N10/P17 in IO leads, N15 at Fpz and P10/N17 in PO leads. In addition the results here also replicate more recent findings, i.e. Todd et al. (2014), of longer latency effects, most notably an N42/P52 deflection in fronto-central leads. A statistical analysis of these effects shows distinct patterns for the short and long-latency effects. The short-latency responses exhibit a contralateral dominance effect, most strongly evident in the posterior P10. The long-latency effects, including the N1 and P2, show a well-defined left ear and right hemisphere advantage. Source analysis of these responses suggests that the short-latency effects may be mediated by a contralateral projection to cerebellum, while the long-latency effects a bilateral projection to right cingulate cortex. Although the non-deterministic nature of the inverse solution does not allow a unique outcome, source analyses nevertheless provide useful hypotheses concerning possible generators. We discuss these results below in detail.

### 4.1 Short latency projections

Considering first the waves for which there are no auditory correlates, the VEMP-related responses in IO and PO leads, none of these showed any effect of ear of stimulation. Neither was there any evidence of an eye bias in the IO leads, nor a neck-side bias in the posterior leads. The only effects observed were in the posterior leads where there was evidence of a contralateral dominance of the initial P10. This was largest for right ear stimulation as measured in the left posterior lead PO7. Although this did not show up as an ear effect, if measured at Iz the right ear response P10 is clearly much larger than for the left.

The absence of a laterality effect in the IO leads is unexpected as the literature on the OVEMP using the standard differential montage is strongly indicative of contralateral preference. It is possible that we did not observe the laterality effect because the subjects' eyes were in neutral gaze. Govender et al. (2009) reported similar amplitudes for AC-evoked ipsilateral and contralateral OVEMPs when subjects' eyes were in the neutral position, with the contralateral response becoming dominant only on up-gaze. The large contralateral OVEMP response is commonly observed with upwards gaze of 20°.

The N15 at Fpz also did not show any ear effects. Given that this wave has been previously identified as being associated with the OVEMP generators (Rosengren et al., 2005; Todd et al., 2008) this shared property with the IO wave responses for the neutral gaze condition is therefore unlikely to be coincidence.

Given the coincidence in latency of the PO contralateral response with that of the contralateral IO waveform it is likely that there is a relationship between these two responses, i.e. that there is a common contralateral vestibular brain source which contributes to both. This is confirmed in the source analysis, which shows that there is a large deep, possibly cerebellar, source co-active with the ocular sources and with the N15. The strong asymmetry observed for right ear stimulation provides an explanation for the laterality effects observed and for the responses being larger with right ear stimulation (although not significant in this case). The appearance of a large contralateral cerebellar source is consistent with the modelling of short-latency VsEPs by Todd et al. (2008).

### 4.2 Long latency projections

Turning now to the longer latency waves measured at central locations, i.e. the N42/P52, N1 and P2, in all cases a very definite ear preference is found. We have recently presented evidence (Todd et al., 2014), that this waveform is generated by vestibular afferents, when stimuli supra-threshold for vestibular receptors are used. In all cases except one, the preference is for left ear over right, the exception being the peak–peak N42/P52 measured at FCz, where there is a right ear preference, although this is a relatively weak effect. Given that the vestibular reflex contributions are symmetrical with respect to ear, as noted above, then we must assume that the ear preference is central, rather than peripheral, i.e. it is a reflection of a central projection dominance from one side, rather than the activation of the otolith organ from that side.

In the case of the N1 and P2 measured in lateral positions, the ear effect interacted with intensity such that the left central-projection dominance was only manifest at the supra-threshold intensity. At supra-threshold intensity the left vs. right amplitude was 3.3 vs. 2.6 μV compared with 1.6 vs. 1.4 μV at sub-threshold intensity. Thus, the left central-projection dominance almost certainly can be attributed to an additional vestibular effect, rather than the previously observed auditory effect (Hine and Debener, 2007).

Of particular relevance to the above is that both N1 and P2 in the lateral positions showed a well-defined laterality effect, so that the left ear central-projection dominance was a contralateral one to the right hemisphere, and thus could be interpreted as a right hemisphere dominance. Some caution is needed in making this conclusion as, if coded by electrode rather than laterality, there was no effect, i.e. overall the mean amplitude in left and right electrodes was the same. However, there was also an interaction between ear and electrode so that the contralaterality effect was only apparent for the projection from left ear to right hemisphere. The above interpretation is strengthened by the source analyses which indicated that for N42, N1 and P2 waves the cingulate source contributed significantly, in addition to temporal cortex sources, and this was clearly lateralised to right hemisphere for the N1. A vestibular cingulate area is consistently indicated in imaging studies and in more recent meta-analyses (Lopez and Blanke, 2011; Lopez et al., 2012).

In the context of the previous literature our finding of evidence for a dominant projection to the right hemisphere is consistent with earlier studies (Dieterich et al., 2003), but it does not support this being an ipsilateral projection, in contrast to the findings of Schlindwein et al. (2008). This difference might be explained by the fact that we have used an acoustic otolithic stimulus, rather than a galvanic or caloric one which primarily activates canals. However, more recent meta-analyses of vestibular imaging studies do not fully support the view that vestibular cortical projections are primarily ipsilateral (Lopez et al., 2012), and other electrophysiological studies in animal models suggest a primarily contralateral projection (Ebata et al., 2004). Thus our data showing a contralateral otolithic projection to the right hemisphere, over and above the previously observed effect for AEPs is very plausible.

### 4.3 Auditory vs. vestibular projections

In healthy subjects the use of AC sound activates cochlear as well as vestibular receptors. In the modelling process for two pairs of regional sources similar solutions were found in all cases, strongly implicating the dominance of auditory cortex plus a contribution from the eyes. In the sub-threshold case the ocular contribution might seem surprising, but given that we did not apply any additional filtering before the analysis, and that the subjects were watching a movie to avoid alpha contamination, the presence of ocular drift in the recordings should be expected. For right ear stimulation there was a complete divergence in the behaviour of the additional sources for the sub- and supra-threshold conditions, with clear evidence of cingulate and cerebellar sources supra- but not sub-threshold. This was not so for left ear stimulation. As noted above though it is very likely that the VEMP threshold is an overestimate of the receptor threshold. McCue and Guinan (1994) found a rate threshold in cat vestibular afferents of 90 dB SPL for 50 ms tones, with a phase-locking threshold 10 dB lower. Combined with the appropriate psychophysical correction for short tone bursts, e.g. Meddis and Lecluyse (2011), this places the receptor rate threshold at near 70 dB SL, about 12 dB below the SLs of our VEMP threshold. It is quite possible, therefore, that we were seeing vestibular effects below VEMP threshold.

In carrying out an analysis of the temporal components we observed some changes in the patterns of activation, most particularly the radial component which exhibited a distinct second peak at about 100 ms, labelled T100. This component, although weakly present contralaterally in sub-threshold conditions, is noticeably more prominent and bilaterally present in the supra-threshold conditions and suggestive of a vestibular projection to temporal lobe, consistent with the imaging studies (Lopez et al., 2012). When viewed directly in T7 and T8 leads, a peak at about 100 ms can be observed (Fig. 2 c and d), but this peak is much more prominent when an average reference is employed, hence its strong appearance in the source analysis which is based on the average reference. A vestibular radial temporal component has been suggested previously for short-latency VsEPs, which is measureable in T8 (McNerney et al., 2011). It is thus quite plausible that a vestibular temporal lobe projection could extend to longer latencies. In the auditory literature a late radial component has been referred to as the “T-complex” (Hine and Debener, 2007), but T-complex studies often use high intensity stimuli, e.g. Connolly (1993) who used AC stimuli of 70 and 90 dB SL. It is also quite possible, therefore, that vestibular contributions to the T-complex have been hitherto unrecognized.

Previously we speculated that the cingulate component activated by vestibular receptors could contribute to the affective quality of sound when above vestibular threshold (Todd et al., 2014). However, the presence of a vestibular component in cortical potentials from the temporal lobe, hitherto considered purely cochlear in origin, also raises the possibility that acoustic activation of the otolith organs could contribute to auditory discrimination. There is now a growing literature which provides evidence of a central vestibular–auditory interaction which allows vestibular inputs to improve temporal and spatial aspects of hearing (Emami and Daneshi, 2012; Brimijoin and Akeroyd, 2012; Probst and Wist, 1990) and to contribute to speech perception and in metrical aspects of musical perception (Emami et al., 2012; Phillips-Silver and Trainor, 2008). Given the well-established cross-over from vestibular to auditory pathways at the level of the brainstem (e.g. Barker et al., 2012) and thalamus (Roucoux-Hanus and Boisacq-Schepens, 1977; Blum et al., 1979), vestibular effects at the level of temporal cortex should be expected, especially as activation of superior temporal lobe is consistently indicated in vestibular imaging studies (Lopez et al., 2012).

## Figures and Tables

**Table 1:** TTCs for 1, 2, 3 and 4 pair models (interval 7–235 ms) at max intensity.

Model	RV	Ear	Source	*X*	*Y*	*Z*	Origin	BA or region
1 pair	7.8	L	RS1&2	±39	−15	11	Ins	13/40
	7.8	R	RS1&2	±36	−13	10	Ins/STG/TTG	13/41
2 pairs	3.2	L	RS1&2	±39	59	−36	EOM + RCD	13/41/22
			RS3&4	±43	−18	8	Ins/STG/TTG	
	3.3	R	RS1&2	±35	53	−32	EOM + RCD	
			RS3&4	±39	−18	11	Ins/STG/TTG	13/41
3 pairs	2.4	L	RS1&2	±39	58	−38	EOM + RCD	
			DP1&2	±4	4	40	CG/MFG	24/32
			RS3&4	±52	−18	3	STG/Ins	22/41/21/13
	2.1	R	RS1&2	±36	60	−36	EOM + RCD	
			DP1&2	±4	−6	38	CG	24/31
			RS3&4	±51	−16	4	STG/Ins	22/41/13/21
4 pairs	1.8	L	RS1&2	±39	57	−38	EOM + RCD	
			DP1&2	±15	−7	41	CG	24/32
			RS3&4	±54	−14	3	STG/PCG	22/21/41/6
			DP3&4	±12	−77	−21	Cerebellum	Posterior lobe/declive
	1.5	R	RS1&2	±34	61	−37	EOM + RCD	
			DP1&2	±4	2	41	CG/MFG	24/32
			RS3&4	±54	−14	3	STG/PCG	22/21/41/6
			DP3&4	±23	−53	−25	Cerebellum	Anterior lobe/dentate

Abbreviations: Brodmann Area (BA), Cingulate Gyrus (CG), Dipole (DP), Extra Ocular Muscles (EOM), Insula (Ins), Left (L), Medial Frontal Gyrus (MFG), Precentral Gyrus (PCG), Retinal Corneal Dipole (RCD), Regional Source (RS), Right (R), Superior Temporal Gyrus (STG), Transverse Temporal Gyrus (TTG), Talairach-Tournoux Coordinates (TTC).

**Table 2:** TTCs for 1, 2, 3 and 4 pair models (interval 7–235 ms) at −12 dB re V_T_.

Model	RV	Ear	Source	*X*	*Y*	*Z*	Origin	BA or Region
1 pair	16.6	L	RS1&2	±43	−19	19	Ins	13/40
	11.3	R	RS1&2	±40	−18	11	Ins/STG/TTG	13/41
2 pairs	4.1	L	RS1&2	±33	62	−36	RCD	
			RS3&4	±43	−20	10	Ins/STG/TTG	13/41/22
	6.1	R	RS1&2	±36	63	−31	RCD	
			RS3&4	±47	−23	3	STG/Ins/MTG	22/13/41/22
3 pairs	3.2	L	RS1&2	±34	61	−37	RCD	
			DP1&2	±12	−16	38	CG	24/31
			RS3&4	±53	−17	5	STG/TTG	22/41/21/13
	4.6	R	RS1&2	±36	62	−33	RCD	
			DP1&2	±42	11	40	MFG/PCG	9/8/6
			RS3&4	±46	−22	0	STG/Ins	22/13/21
4 pairs	2.8	L	RS1&2	±33	61	−37	RCD	
			DP1&2	±10	−5	40	CG	24/31
			RS3&4	±56	−18	1	STG/MTG	22/21/41
			DP3&4	±20	−72	−19	Cerebellum	Posterior lobe/declive
	–	R	RS1&2	–	–	–	–	–
			DP1&2	–	–	–	–	–
			RS3&4	–	–	–	–	–
			DP3&4	–	–	–	–	–

Abbreviations: Brodmann Area (BA), Cingulate Gyrus (CG), Dipole (DP), (Ins), Left (L), Medial Frontal Gyrus (MFG), Middle Temporal Gyrus (MTG), Precentral Gyrus (PCG), Retinal Corneal Dipole (RCD), Regional Source (RS), Right (R), Superior Temporal Gyrus (STG), Transverse Temporal Gyrus (TTG), Talairach-Tournoux Coordinates (TTC).

**Table 3:** Source current strengths (nA) for 4-pair model contributing to supra-threshold conditions for both left vs. right ear stimulation. Large sources are indicated in bold.

		1st Lobe		2nd Lobe		4th Lobe		5th Lobe		6th Lobe (110 ms)		6th Lobe (172 ms)	
		Left ear	Right ear	Left ear	Right ear	Left ear	Right ear	Left ear	Right ear	Left ear	Right ear	Left ear	Right ear
Ocular	LH	8	6	**12**	6	3	4	1	2	3	4	2	3
	RH	6	5	**11**	9	6	3	2	1	2	2	5	1
Cerebellar	LH	9	**13**	2	**17**	3	7	2	1	1	1	1	3
	RH	7	1	2	1	1	5	0	6	7	7	1	3
Termporal (tangential component)	LH	1	1	0	2	3	1	6	**11**	1	1	**11**	**11**
	RH	2	1	1	1	2	3	**15**	**10**	4	3	**14**	8
Temporal (radial component)	LH	0	5	0	4	1	2	5	10	7	**13**	6	6
	RH	1	3	2	3	1	1	4	2	**11**	7	10	9
Cingulate	LH	2	1	3	7	3	**6**	2	4	0	0	10	6
	RH	1	6	1	0	**8**	2	**11**	**10**	4	6	5	8

Abbreviations: LH left hemisphere, RH right hemisphere.
